# CT colonography followed by elective surgery in patients with acute diverticulitis: a radiological-pathological correlation study

**DOI:** 10.1007/s00261-020-02690-5

**Published:** 2020-08-03

**Authors:** Nicola Flor, Perry J. Pickhardt, Giovanni Maconi, Silvia Panella, Monica Falleni, Valeria Merlo, Giovanni Di Leo

**Affiliations:** 1 Unità Operativa di Radiologia, L. Sacco University Hospital, ASST Fatebenefratelli Sacco, Via Giovanni Battista Grassi, 74, 20157 Milan, Italy; 2 Department of Radiology, School of Medicine & Public Health, University of Wisconsin, E3/311 Clinical Science Center, 600 Highland Ave, Madison, WI 53792-3252 USA; 3 Gastroenterology Unit, Department of Biomedical and Clinical Sciences, L. Sacco University Hospital, Milan, Italy; 4 Unità Operativa Radiologia Diagnostica e Interventistica, ASST Santi Paolo e Carlo, Presidio San Paolo, Milan, Italy; 5 Unità Operativa di Anatomia Patologica, Azienda Ospedaliera San Paolo, Via di Rudinì 8, 20142 Milan, Italy; 6 Dipartimento di Scienze della Salute, Università degli Studi di Milano, Via di Rudinì 8, 20142 Milan, Italy; 7 Postgraduation School in Radiodiagnostics, Facoltà di Medicina e Chirurgia, Università degli Studi di Milano, Via Festa del Perdono 7, 20122 Milan, Italy; 8 Unità di Radiologia, IRCCS Policlinico San Donato, San Donato Milanese, Italy; 9 Dipartimento di Scienze Biomediche della Salute, Università degli Studi di Milano, Piazza E. Malan, 20097 San Donato Milanese, Italy

**Keywords:** Diverticular disease, Acute diverticulitis, Colon, Abdominal CT, CT colonography, Surgery

## Abstract

**Purpose:**

To perform a radiologic-pathologic correlation analysis of sigmoid colon in patients undergoing pre-operative CT Colonography (CTC) after an episode of acute diverticulitis (AD).

**Methods:**

Fifty-nine consecutive patients (31/28 M/F; 58 ± 13 years) underwent CTC 55 ± 18 days after AD, 8 ± 4 weeks before surgery. Thirty-seven patients (63%) underwent conventional abdominal CT at time of AD. An experienced blinded radiologist retrospectively analyzed all images: disease severity was graded according to the Ambrosetti classification on conventional CT and according to the diverticular disease severity score (DDSS) on CTC. A GI pathologist performed a dedicated analysis, evaluating the presence of acute and chronic inflammation, and fibrosis, using 0–3 point scale for each variable.

**Results:**

Of 59 patients, 41 (69%) had at least one previous AD episode; twenty-six patients (44%) had a complicated AD. DDSS was mild-moderate in 34/59 (58%), and severe in 25/59 (42%). All patients had chronic inflammation, while 90% had low-to-severe fibrosis. Patients with moderate/severe fibrosis were older than those with no/mild fibrosis (61 ± 13 versus 54 ± 13). We found a significant correlation between DDSS and chronic inflammation (*p *= 0.004), as well as DDSS and fibrosis (*p *= 0.005). Furthermore, fibrosis was correlated with complicated acute diverticulitis (*p *= 0.0.27), and with age (*p *= 0.067). At multivariate analysis, complicated diverticulitis was the best predictor of fibrosis (odds ratio 4.4). Patient age and DDSS were other independent predictors.

**Conclusion:**

DDSS-based assessment on preoperative CTC was a good predictor of chronic colonic inflammation and fibrosis. In addition, the presence of complicated diverticulitis on CT during the acute episode was most predictive of fibrosis.

## Introduction

Diverticular disease represents the most common disease affecting the colon in the Western world [1]. An estimated 5–25% of patients with colonic diverticulosis will develop abdominal symptoms (symptomatic uncomplicated diverticular disease) and eventually complications as acute diverticulitis (AD) in their lifetime [2]. Treatment of AD depends on the severity of the event: following recent guidelines [3, 4], therapeutic strategies are more and more conservative, and emergency surgery is indicated only in case of peritonitis [5]. However, diverticular disease remains the leading indication for elective colon resection [6, 7].

Although the appropriate diagnostic work-up after recovery from an episode of AD is still controversial [8, 9], CT colonography (CTC) is the most accurate radiologic test for evaluating the colon and it is proven to be a safe and accurate exam in the follow-up of AD [7, 10]. In patients who have clinically recovered from an episode of AD, CTC gives detailed information on the extent of diverticular disease; the main CTC findings in cases of severe diverticular disease include marked colonic wall thickening, typically involving a long segment, associated with severe luminal stenosis [11, 12]. These CTC findings are key findings for the DDSS classification of disease severity based on CTC [13]. In particular, DDSS has proved to have predictive value for the course of the disease [14], and marked wall thickening (DDSS score of 4) generally signifies the presence of chronic inflammation with fibrosis.

To investigate our hypothesis, we compared radiological and pathological findings in a cohort of patients who have recovered from an episode of acute diverticulitis who underwent imaging follow-up with CTC and subsequent elective surgery.

## Materials and methods

### Study population

A non-specific written informed consent for the use of personal anonymized data in retrospective studies was obtained from all patients. Inclusion criteria were: a documented episode of AD (with availability of records of Emergency Department discharge records), a CTC examination performed after that AD episode, and subsequent elective surgery for diverticular disease. Exclusion criteria included: patients affected by Crohn’s disease or ulcerative colitis, previous colonic surgery, or colorectal cancer.

The final study sample included a series of 59 patients (31 males and 28 females), with a mean age of 58 ± 13 years. All CTC examinations and surgery were performed in our Hospital [Blinded], from 2018 and 2019. Among the 59 total patients other than a CTC, 37/56 (63%) patients had also had a conventional abdominal CT at the time of AD episode at emergency admission; the remaining 22 (37%) had either a clinical visit alone (8/22) or a clinic visit in association with ultrasound or conventional radiography (14/22). For all patients, it was possible to assess the severity of disease at the time of the AD episode was assessed, using the classification system proposed by Ambrosetti as complicated or not complicated [15].

### CT and CTC protocol

Abdominal CT was performed at the time of emergency department admission. All examinations were performed using a 16-row unit (BrightSpeed, General Electric Healthcare, Waukesha, WI, USA). After an unenhanced CT scan, a second scan was acquired 60–70 s after intravenous injection of 100 mL of non-ionic iodinated contrast material (Iomeron® 400, Bracco Imaging SpA, Milan, Italy) followed by 50 mL of saline flush, both at a flow rate of 3 mL/s, thus obtaining images during the portal venous phase. This portal venous phase is particularly useful in evaluating the severity of the event; in particular it enhances the presence of abscesses, facilitating the diagnosis of complicated acute diverticulitis.

The CT imaging protocol was as follows: gantry rotation time 0.8 s; slice thicknes, 1.25 mm; pitch 1.375; reconstruction interval 1.25 mm; tube voltage 120 kVp (140 in obese patients), modulated tube current from 80 to 440 mA.

CTC exams were performed a mean of about 2 months (55 ± 18 days) after AD episode. Standard bowel preparation consisted of one sachet of mild oral laxative (Movicol; Norgine Italia srl; Milan, Italy) after breakfast, lunch and dinner for the 3 days before CTC with water-soluble iodinated contrast material (diatrizoate) taken 3 h before CTC examination for fecal tagging. Colonic distention was achieved with automated carbon dioxide (CO_2_) insufflation. The patient was placed in the left decubitus position for the initial 1.5 L of CO_2_, followed by the supine and right lateral decubitus positions for up to 1 L each, followed by the supine position. All the follow-up CTC examinations were performed using a 64-row scanner (LightSpeed; General Electric Medical Systems, Milwaukee, Wisconsin, USA). The CTC acquisition protocol was as follows: gantry rotation time 0.5 s; slice thickness 1.25 mm; table speed 27.5 mm/s; pitch 1.375; reconstruction interval 1 mm; tube voltage 120 kVp (140 in obese patients); tube current 100 mA (300 in obese patients).

Standard imaging protocol included supine and prone scans; a third additional lateral decubitus scan was performed in 28/59 (47%) to achieve the best distension of the sigmoid colon. In patients not able to lay in the prone decubitus, an alternative lateral scan was performed.

### Image analysis

A radiologist with 15 years of experience in CTC retrospectively re-analyzed all examinations, including conventional abdominal CT performed at the time of AD in the Emergency Department, and the subsequent follow-up CTC examination after recovery. CTC assessment was blinded to the conventional CT findings, as well as to the clinical and pathologic results.

On conventional CT examination, maximum colonic wall thickening and its longitudinal extent along the site of acute inflammation were measured.

For each patient, the severity of disease at follow-up CTC was calculated using the established diverticular disease severity score (DDSS), a four-class classification proposed by Flor et al. [13], based on maximum sigmoid colon wall thickness and minimum lumen diameter, measured from inner-to-inner wall. The reader first selected the series (prone, supine or right lateral decubitus) in which the sigmoid colon was best distended. All the measurements were taken, in millimetres, on 2D multiplanar reformatted images from the best distended series, always on the images perpendicular to the long axis of the colon segment, using standard soft tissue windowing. For the purpose of this study, we assumed DDSS 1 and 2 to represent mild-moderate diverticular disease and DDSS 3 and 4 to represent severe diverticular disease [13]. The same radiologist evaluated the presence of colonic polyps with a diameter ≥ 6 mm.

### Elective surgery and pathology

All patients underwent elective surgery a mean of 8 ± 4 weeks after CTC at our Institution. Elective surgery was performed in an inflammation-free time and therefore postponed until after antibiotic therapy. Preoperative CTC findings were available to the surgeons, who followed the World Gastroenterology Organization surgical guidelines for diverticular disease [16]. The resection always involved the sigmoid colon and was performed using either laparoscopic or open resection techniques according to surgeon preference. The colon segment affected by AD was resected, extending the resection according to the vascular pedicle, severity, and extension of diverticular disease.

Sigmoid colon surgical biopsy sampling was performed on areas of maximum inflammation and bowel wall thickness. Specimens were fixed in formalin; paraffin-embedded samples and 5-µm-thick slides prepared with haematoxylin–eosin-stain were microscopically examined. A pathologist with 15 years of experience in abdominal intestinal diseases analyzed all specimens. Wall thickness was measured on histological slides from mucosal to serosal surface, in the area of greatest thickness. The presence of inflammation and/or fibrosis was recorded. In particular, inflammation was evaluated and classified as acute, chronic, or mixed (both acute and chronic). For each variable (acute inflammation; chronic inflammation; fibrosis), a 4-point scale was used: 0 (absence); 1 (low); 2 (moderate); 3 (severe) [17, 18]. However, for statistical analysis, all these three pathologic outcomes were dichotomized.

### Statistical analysis

Findings of conventional CT and CTC were correlated with the results from pathologic specimen analysis. Being the three pathologic outcomes (acute inflammation, chronic inflammation, and fibrosis) probably correlated among them, one was chosen as primary outcome according to the strength of the mutual association. Once identified the primary outcome, bivariate associations with potential predictors were first investigated using the Chi square test. Then, variables that were found to be associated with the primary outcome were later entered into a multivariate regression analysis. A predicting model was developed using R^2^ and statistical significance for deciding which predictors to keep in the model. Exploratory analysis was performed for secondary outcomes.

## Results

### Patients’ characteristics

Patients’ main characteristics are shown in Table 1. Of 59 patients, 41 (69%) had one or more previous episodes of AD, whereas the remaining 18/59 (36%) were experiencing their first AD episode. Compared to patients with their first AD episode, those with a history of previous episodes of AD had a higher rate of chronic inflammation (73% versus 50%) and a higher rate of fibrosis (54% versus 39%), although these two associations were not significant (*p *≥ 0.083).

**Table 1:** Patients’ main characteristics

Variable	Value
Age (years)	58 ± 13
Male sex (%)	31 (53%)
History of previous AD	41 (69%)
Complicated AD	26 (44%)
C-reactive protein (mg/L)	
0–50	15 (25%)
50–100	15 (25%)
100–150	6 (10%)
Unknown	23 (40%)
Maximum colonic wall thickening (mm)^a^	12 ± 3
Longitudinal extent (mm)^a^	107 ± 44
DDSS	
Mild-moderate	34 (58%)
Severe	25 (42%)
Acute inflammation	
Absent-mild	41 (69%)
Moderate-severe	18 (31%)
Chronic inflammation	
Absent-mild	20 (34%)
Moderate-severe	36 (66%)
Fibrosis	
Absent-mild	30 (51%)
Moderate-severe	29 (49%)

*AD* acute diverticulitis, *DDSS* diverticular disease severity score

^a^On conventional computed tomography examination

Following classification proposed by Ambrosetti [15], 26 of 59 patients (44%) had a complicated AD: 6 patients had fistulas, 18 had an abscess, and 17 had extraluminal air. C-reactive protein (mg/L) was available in 36 patients: it was 0–50 in 15, 50–100 in 15, 100–150 in 6.

CTC examination revealed at least one polyp ≥ 6 mm in 10/59 (17%) patients.

### Severity of diverticular disease based on DDSS

On conventional CT examination, maximum colonic wall thickening and its longitudinal extent were 12 ± 3 mm and 107 ± 44 mm, respectively. Based on CTC, DDSS was mild-moderate in 34/59 (58%) patients, and severe in 25/59 (42%). The latter were slightly but not significantly older the former (60 ± 13 versus 55 ± 13, *p *= 0.144).

### Pathological analysis and selection of the primary outcome

**Fig. 1:**
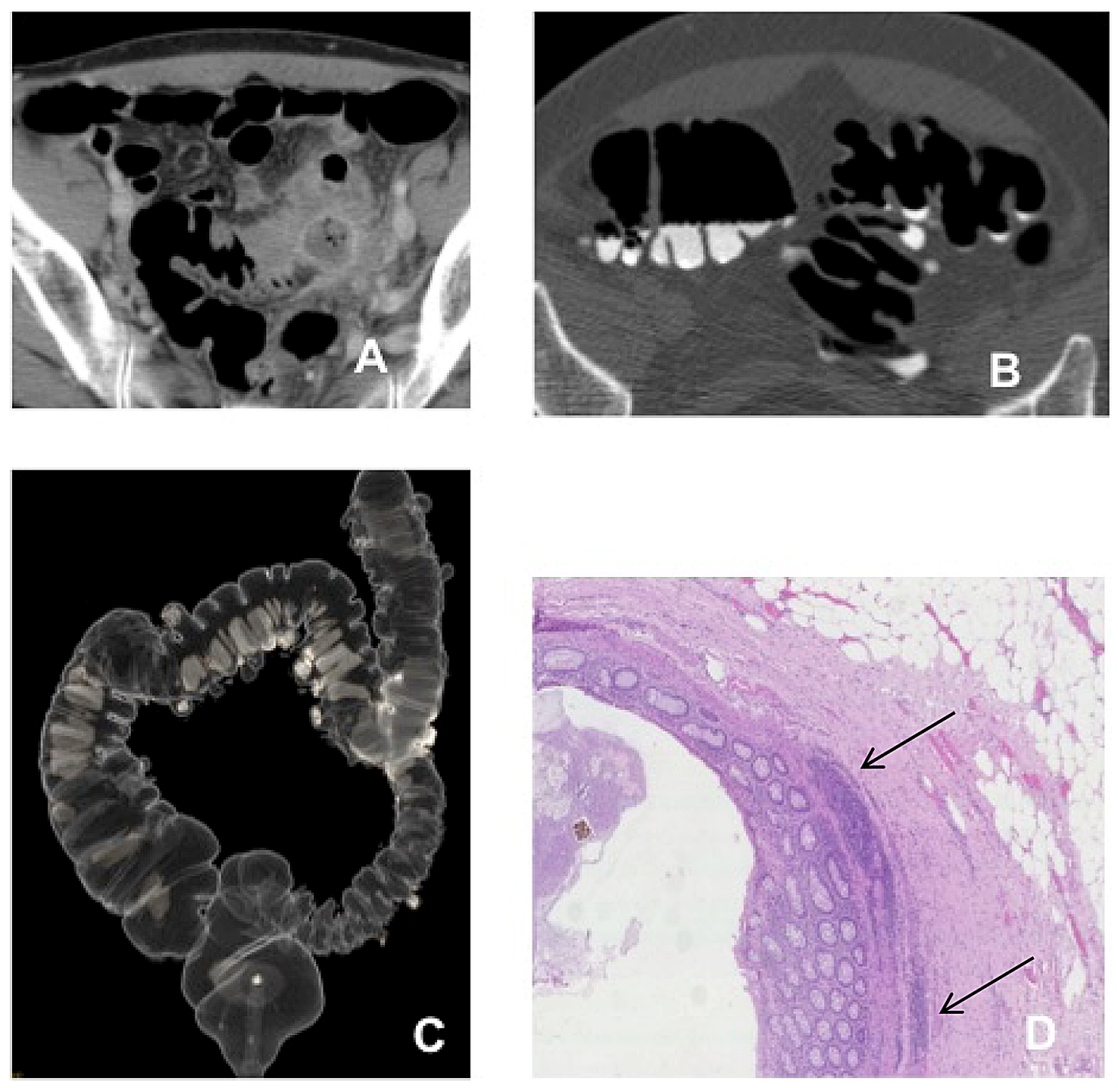
Images in a 59-year-old-man who underwent elective surgery after recovery from acute diverticulitis. Axial two-dimensional (2D) supine conventional computed tomography (CT) **a** shows acute diverticulitis in the presence of sigmoid colon wall thickening, mesenteric fascial thickening and small abscess (complicated AD). Axial 2D supine computed tomography colonography (CTC) image **b** and DCBE CTC like-view **c** images showing sigmoid colon diverticula without wall thickening or significant lumen stenosis (diverticular disease severity score [DDSS] 1). In this whole-thickness section of the colonic wall (**d**), only scarce fibrosis and focal chronic inflammation (arrows) is appreciable

**Fig. 2:**
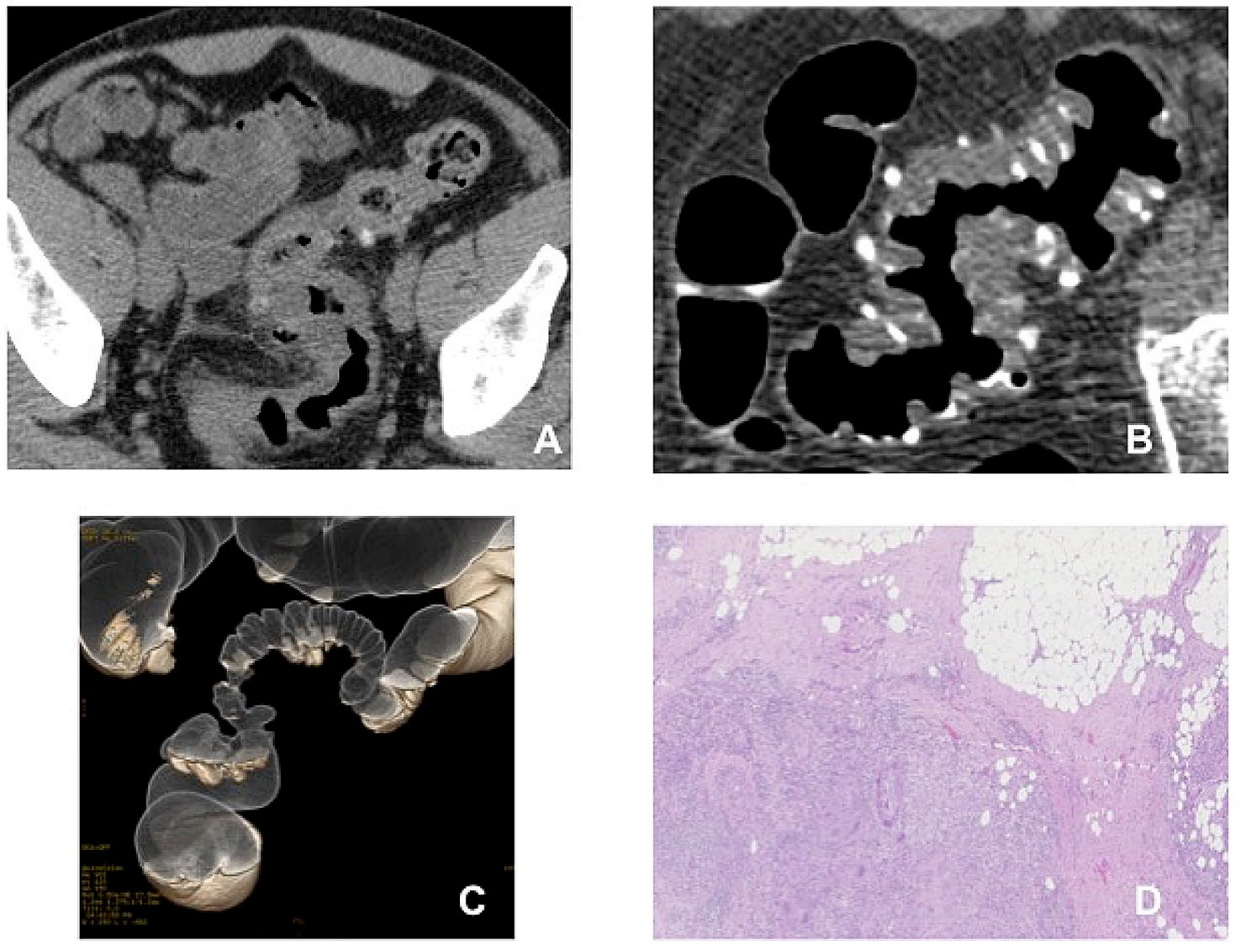
Images in a 49-year-old-man who underwent elective surgery after recovering from acute diverticulitis. Axial two-dimensional (2D) supine conventional computed tomography (CT) **a** shows acute diverticulitis in the presence of sigmoid colon wall thickening, inflamed diverticula, mesenteric fat stranding and mesenteric fascial thickening (uncomplicated AD). Axial 2D supine computed tomography colonography (CTC) image (**b**) and DCBE CTC like-view **c** images showing sigmoid colon diverticula in the presence of severe diffuse wall thickening and severe lumen stenosis (diverticular disease severity score [DDSS] 4). Heavy, dense fibrosis intermingled with inflammatory cells is appreciable at histology (**d**). Note fibrotic bands in perivisceral fat tissue around large bowel walls

Pathology confirmed the diagnosis of diverticular disease in all the 59 patients. Any differential diagnosis was considered, and in particular, no patients had superimposed cancer or Inflammatory Bowel Disease. Distribution of acute inflammation, chronic inflammation, and fibrosis are reported in Table 2. All patients had chronic inflammation, while 90% had from low to severe fibrosis. Examples are shown in Figs. 1 and 2.

**Table 2:** Result of pathologic analysis crossed with DDSS and the Ambrosetti scores

	Acute inflammation (moderate/severe)	Chronic inflammation (moderate/severe)	Fibrosis
Complicated AD			
DDSS 1-2	3 (5%)	8 (14%)	8 (14%)
DDSS 3-4	7 (12%)	11 (19%)	9 (15%)
Not complicated AD			
DDSS 1-2	3 (5%)	10 (17%)	5 (9%)
DDSS 3-4	5 (9%)	10 (17%)	7 (12%)

Data are counts and percentages

*AD* acute diverticulitis

Chronic inflammation was substantially, yet not significantly associated to acute inflammation: chronic inflammation was recorded in 24 of 41 (59%) patients without acute inflammation, in 15 of 18 (83%) patients with acute inflammation (*p *= 0.064). Instead, chronic inflammation was strongly and significantly associated to fibrosis: chronic inflammation was recorded in 14 of 30 (47%) patients without fibrosis, in 25 of 29 (86%) patients with fibrosis (*p *= 0.001). Thus, fibrosis was selected as primary outcome.

### Relationship between radiological and pathological findings

At bivariate analysis, fibrosis was positively associated with DDSS (*p *= 0.050), complicated diverticulitis (*p *= 0.027), abscess (*p *= 0.021), maximum wall thickness (*p *= 0.026), and minimum lumen diameter (*p *= 0.030). Moreover, patients with moderate/severe fibrosis were older than those with no or mild fibrosis (61 ± 13 versus 54 ± 13, respectively – *p *= 0.069). The extent of the pathological wall thickening did not correlate with fibrosis (*p *= 0.513).

At multivariate linear regression (Table 3), complicated diverticulitis diagnosed at time of the acute event was the most predictive variable for fibrosis, with an odds ratio of 4.4. Other relevant/significant predictors were patient age and DDSS.

**Table 3:** Results of the multivariate regression analysis

	T	E.S.	Wald	Df	Sign.	Odds ratio
Complicated AD	1.478	0.625	5.597	1	0.018	4.385
DDSS 3-4	0.747	0.594	1.582	1	0.208	2.111
Age	0.047	0.024	3.958	1	0.047	1.049
Constant	− 3.727	1.495	6.211	1	0.013	0.024

*AD* acute diverticulitis, *df* degree of freedom

At exploratory analysis of chronic inflammation, we observed a strong association with DDSS (*p *= 0.004). In particular, patients with DDSS 2 had a high prevalence of mild and moderate chronic inflammation, whereas patients with DDSS 3 had only few cases of mild chronic inflammation. Among patients with DDSS 4, we found only cases of moderate and severe chronic inflammation.

## Discussion

As main results of our study, we found a strong correlation between chronic inflammation and fibrosis, and we demonstrated a significant predictive value of the CTC-based score (i.e., the DDSS) for chronic inflammation and fibrosis. CTC is able to confirm the presence of colonic diverticula, to describe their distribution for the entire colon, provide for stratification of disease severity, and to diagnose complications such as abscesses and fistulas [19]. The possibility of predicting the presence of chronic inflammation and fibrosis further enhances the role of CTC in following-up patients recovered from acute diverticulitis. The CTC-based data can better inform clinicians and surgeons as to the most appropriate management. In fact, CTC diagnosis of chronic inflammation and fibrosis could help to explain the ineffectiveness of medical treatment in some patients, suggesting the need for surgical management.

Moreover, results from our radiological-pathological comparison shed more light on the predictive value of CTC reported in previous studies [13, 14]. The correlation found between DDSS, a CTC severity score score based on wall thickening and lumen narrowing, and fibrosis has strong similarities in the paper of Melchior et al. [18]. In fact, evaluating the role of Magnetic resonance for fibrosis assessment in rats, they found a positive relationship between histologic score of fibrosis and colonic wall thickness, luminal narrowing, and mural high signal intensity in T2-weighted sequences.

Limiting analysis to fibrosis, we found that complicated diverticulitis diagnosed at time of the acute event was the best positive predictive variable. This result is analogous to previous studies that reported a higher risk of recurrence for patients with complicated diverticulitis diagnosed by conventional CT at the time of the acute initial episode [20–23].

Translating these results to routine practice, imaging follow-up with CTC should be considered for patients aged 50 years and older with complicated acute diverticulitis. Another interesting result was the lack of correlation between history of previous episodes of acute diverticulitis and fibrosis. In fact, this result seems to reinforce recent trends that no longer consider the number of previous episodes of acute diverticulitis as an indication for elective surgery [3].

Some limitations of our study should be mentioned. First, we haven’t investigated the potential relationship between dietary fiber and colonic wall thickness [24–26]; to do that we should have used one or more dedicated specific dietary questionnaires, assessing also lifelong low residue diet of patients. Moreover, as a general advice by general practicioners and gastroenterologists, patients with diverticulosis are encouraged to eat fibers, but not immediately after episodes of acute diverticulitis. Therefore, whether the amount of dietary fibers might have affected colonic wall thickness and DDSS is very difficult to demonstrate, although unlikely, given the fibrosis and lack of elasticity.

Second, the selection of patients based on surgical resection could introduce selection bias. Actually, there is a balanced distribution of chronic inflammation and fibrosis among the patients, due to the fact that surgical criteria were different. In particular, among patients who underwent elective surgery there were also young healthy patients with a unique previous episode of acute diverticulitis,. Moreover, in a previous paper [14] focused on the same topic we had the opportunity to test the predictive value of DDSS in a cohort including surgically and non-surgically patients.

In conclusion, we have demonstrated that CTC is a good predictor of chronic inflammation and fibrosis, and that conventional CT demonstrating complicated diverticulitis at the initial episode is a good predictor of fibrosis. Therefore, it seems reasonable to choose CTC in following up patients after recovery from an episode of acute diverticulitis, paying particular attention to those with complicated initial diverticulitis.

## Abbreviations

AD: Acute diverticulitis
CTC: Computed tomography colonography
